# Enhanced Production of D-Lactate in Cyanobacteria by Re-Routing Photosynthetic Cyclic and Pseudo-Cyclic Electron Flow

**DOI:** 10.3389/fpls.2019.01700

**Published:** 2020-01-31

**Authors:** Tiago Toscano Selão, Jasmin Jebarani, Nurul Aina Ismail, Birgitta Norling, Peter Julian Nixon

**Affiliations:** ^1^ School of Biological Sciences, Nanyang Technological University, Singapore, Singapore; ^2^ Department of Life Sciences, Imperial College London, London, United Kingdom

**Keywords:** cyanobacteria, cyclic electron flow, Mehler-like reaction, metabolic engineering, D-lactate

## Abstract

Cyanobacteria are promising chassis strains for the photosynthetic production of platform and specialty chemicals from carbon dioxide. Their efficient light harvesting and metabolic flexibility abilities have allowed a wide range of biomolecules, such as the bioplastic polylactate precursor D-lactate, to be produced, though usually at relatively low yields. In order to increase photosynthetic electron flow towards the production of D-lactate, we have generated several strains of the marine cyanobacterium *Synechococcus* sp. PCC 7002 (Syn7002) with deletions in genes involved in cyclic or pseudo-cyclic electron flow around photosystem I. Using a variant of the *Chlamydomonas reinhardtii* D-lactate dehydrogenase (LDH^SRT^, engineered to efficiently utilize NADPH *in vivo*), we have shown that deletion of either of the two flavodiiron *flv* homologs (involved in pseudo-cyclic electron transport) or the Syn7002 *pgr5* homolog (proposed to be a vital part of the cyclic electron transport pathway) is able to increase D-lactate production in Syn7002 strains expressing LDH^SRT^ and the *Escherichia coli* LldP (lactate permease), especially at low temperature (25°C) and 0.04% (v/v) CO_2_, though at elevated temperatures (38°C) and/or high (1%) CO_2_ concentrations, the effect was less obvious. The Δ*pgr5* background seemed to be particularly beneficial at 25°C and 0.04% (v/v) CO_2_, with a nearly 7-fold increase in D-lactate accumulation in comparison to the wild-type background (≈1000 vs ≈150 mg/L) and decreased side effects in comparison to the *flv* deletion strains. Overall, our results show that manipulation of photosynthetic electron flow is a viable strategy to increase production of platform chemicals in cyanobacteria under ambient conditions.

## Introduction

There is currently great interest in exploring the use of photoautotrophs such as the prokaryotic cyanobacteria to produce industrially important molecules including biofuels, specialty chemicals and pharmaceutical/nutritional products (Woo, 2017). Cyanobacteria use sunlight to drive the biosynthesis of organic molecules from water and carbon dioxide during the process of oxygenic photosynthesis. Compared to commonly used platforms, such as the yeast *Saccharomyces cerevisiae* and the bacterium *Escherichia coli*, cyanobacteria do not require an organic carbon feedstock and use solar energy for growth, important advantages for the development of a low-cost, carbon-neutral production system.

Rapid progress is being made to develop the necessary genetic tools to manipulate cyanobacteria, especially the model cyanobacteria *Synechococcus* sp. PCC 7002 and *Synechocystis* sp. PCC 6803 [reviewed in Sun et al. (2018)], and a wide range of metabolic engineering experiments have been performed to alter central carbon metabolism to improve the yields of diverse target molecules [reviewed in (Xiong et al., 2017)].

However, less work has been directed at engineering the light reactions of oxygenic photosynthesis, e.g. to enhance the availability of reductive power to drive biosynthetic processes within the cyanobacterial cell. NADPH is produced by ferredoxin:NADP^+^ reductase (FNR) using reduced ferredoxin generated by photosystem I (PSI) (Lea-Smith et al., 2016). Besides being used for CO_2_ fixation in the Calvin-Benson-Bassham cycle, reduced ferredoxin also drives a variety of other reductive processes including nitrogen and sulphur assimilation (Hanke and Mulo, 2013), the reduction of plastoquinone during cyclic electron flow around PSI to generate ATP (Shikanai and Yamamoto, 2017), and the conversion of oxygen to water by flavodiiron (Flv) proteins in a Mehler-like reaction (Helman et al., 2003; Allahverdiyeva et al., 2013). It has been estimated that 15-30% of the electrons coming from the oxidation of water might ultimately be used by Flv to reduce oxygen back to water in so-called pseudocyclic electron flow (Helman et al., 2003).

Cyclic electron flow around PSI in cyanobacteria is currently thought to occur *via* two main routes: the NDH (NADH dehydrogenase-like) pathway, involving a PSI/NDH-1 supercomplex (Gao et al., 2016; Schuller et al., 2019), and the poorly characterized antimycin-sensitive Pgr5 pathway (Yeremenko et al., 2005). In plant chloroplasts, PGR5 is thought to function as a complex with PGRL1 (DalCorso et al., 2008), whereas in cyanobacteria, PGRL1 homologues are absent (Labs et al., 2016). Although NDH and PGR5 play physiologically important roles in cyclic electron flow, it is still unclear whether their roles are direct or indirect (Nandha et al., 2007; Nawrocki et al., 2019). One dramatic feature of the *pgr5* mutant of *Arabidopsis* is an inability to downregulate photosynthetic electron flow, which leads to aberrant over-reduction of the acceptor side of PSI and enhanced photodamage to PSI (Munekage et al., 2002) especially under fluctuating light (Suorsa et al., 2012). In contrast, the *pgr5* null mutant of *Synechocystis* sp. PCC 6803 (hereafter Syn6803) shows more robust growth under these conditions (Allahverdiyeva et al., 2013).

**Figure 1:**
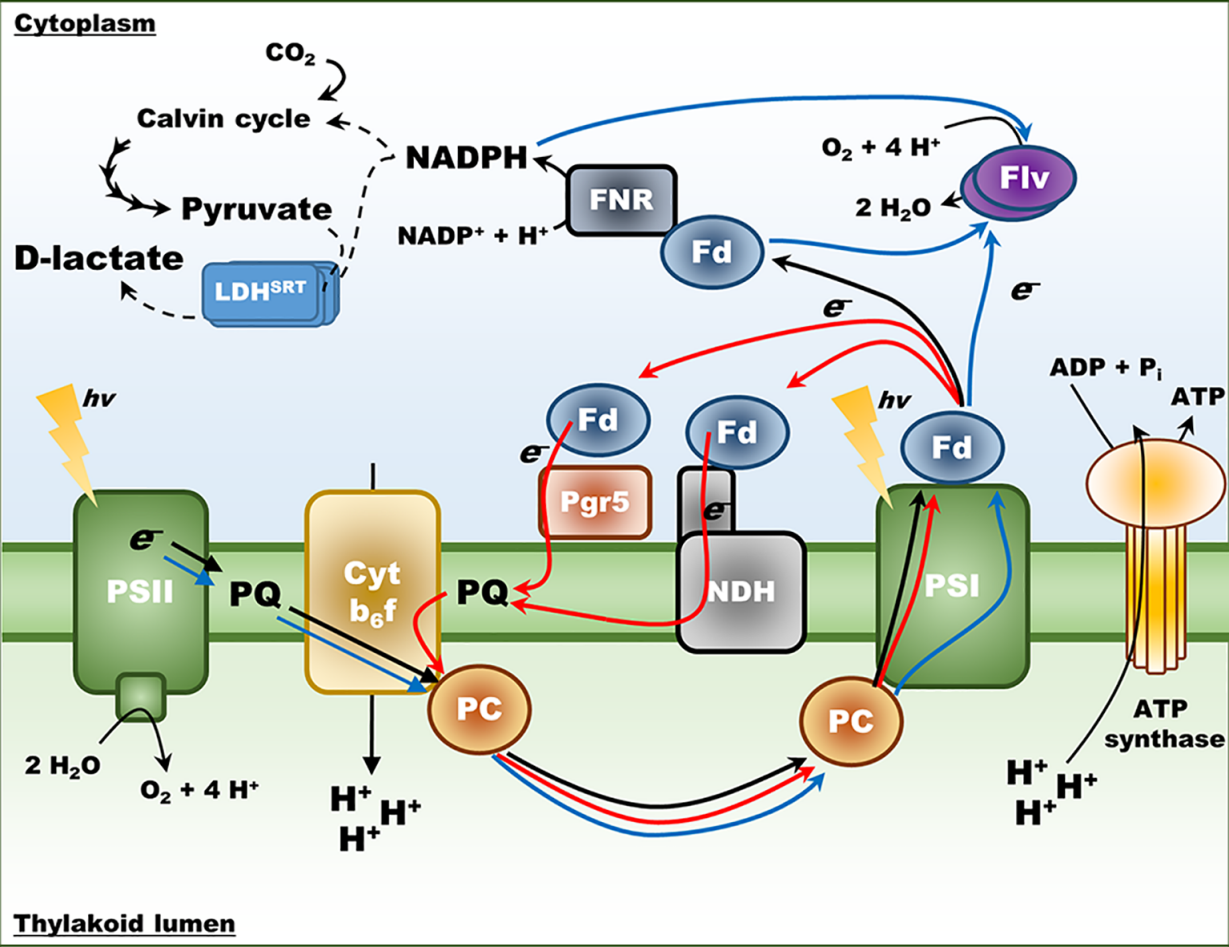
Schematic representation of photosynthetic electron transport in cyanobacteria. Black arrows – Linear Electron Flow (LEF) from water to NADP^+^. Red arrows – Cyclic Electron Flow (CEF), both the pathway involving Pgr5 and the pathway involving NDH. Blue arrows – pseudo-Cyclic Electron Flow (pCET), mediated by the Flv proteins. The D-lactate dehydrogenase variant (LDH^SRT^) is included for reference, converting pyruvate derived from the Calvin cycle into D-lactate and utilizing NADPH as an electron source. PSI, photosystem I; PSII, photosystem II, Fd, ferredoxin; FNR, ferredoxin:NADP^+^ oxidoreductase; PC, plastocyanin; Cyt b_6_f, cytochrome b_6_f.

Here we have tested whether loss of Flv and Pgr5 functions in cyanobacteria, which are predicted to lead to an enhanced reduction state of the ferredoxin and NADPH electron acceptors downstream of PSI, can be exploited to enhance the biosynthesis of molecules dependent on reducing power (**Figure 1**). To do this, we have established a strain of the cyanobacterium *Synechococcus* sp PCC 7002 (henceforth Syn7002) that produces D-lactate *via* the NADPH-mediated reduction of pyruvate and examined D-lactate production in mutants lacking either one or both of the two Flv subunits found in Syn7002 (annotated as Flv1 and Flv3) as well as Pgr5. Our results indicate that manipulation of both these alternative electron transport pathways does indeed improve D-lactate production when cyanobacteria are grown at a lower temperature (25°C) than that optimal for growth and in air-levels of CO_2_. Our work demonstrates that re-routing photosynthetic electron flow is a useful target for metabolic engineering in cyanobacteria.

## Materials and Methods

### Strains, Media, and Growth Conditions

Syn7002 was routinely grown in A+ medium (Stevens et al., 1973) with D7 micronutrients (Arnon et al., 1974), as previously described (Selão et al., 2019). For growth in ambient CO_2_ conditions, cultures were incubated in an FH-1200 growth chamber (HiPoint, Taiwan) and cell growth under 1% CO_2_-enriched air was performed in a 740-FHC LED growth chamber (HiPoint, Taiwan), at a constant light intensity of 250 µmol photons·m^-2^·s^-1^. Strain growth and testing was routinely done in 15 ml cultures grown in upright tissue culture flasks (Corning, part #3056), inoculated at a starting OD_730_ of 0.05, measured in a 1 cm-light path Cary 300Bio (Varian) spectrophotometer using AD7 as blank. For large-scale growth monitoring, OD_730_ was measured in 150 μL of cultures (diluted as needed to OD_730_ < 0.5 with regular AD7) in 96-well plates using a Hidex Sense plate reader (Hidex) and converting to the equivalent OD_730_ by the Cary spectrophotometer, using an in-house derived 70-point calibration curve (R^2^ = 0.9818). Each individual replicate was measured in duplicate. Whole cell spectra were recorded in 96-well plates, using the Hidex Sense plate reader, as described above.

*Escherichia coli* Stellar supercompetent cells, utilized for all cloning steps, were routinely grown in Luria-Bertani (LB) medium, supplemented with appropriate antibiotics as indicated.

### Cloning and Cyanobacterial Transformation

All plasmids (see **Table 1**) were constructed using the pEASY-Uni Seamless Cloning and Assembly Kit (TransGen Biotech, China), according to manufacturer's instructions (unless otherwise specified) and transformed into supercompetent *Escherichia coli* Stellar supercompetent cells (TaKaRa). For a list of primers employed, please consult **Table S1**. Syn7002 transformation was performed as previously described (Selão et al., 2019), with strains selected in AD7-agar [AD7 supplemented with 1.2% (w/v) agar and 1 g/L sodium thiosulfate prior to autoclaving] using antibiotics (50 µg·ml^-1^ spectinomycin, 10 µg·ml^-1^ chloramphenicol, or 50 µg·ml^-1^ erythromycin or combinations thereof) and/or 100 µM acrylate, for constructs targeting the *acsA* locus (Begemann et al., 2013). Full genomic segregation was tested by colony PCR using specific primers (see **Table S1**). All plasmid sequences were confirmed by Sanger sequencing.

**Table 1:** List of plasmids used or generated in this study.

Plasmid name	Origin	Features
**pUC19**	Stratagene	*E. coli* cloning vector
**pGEM-T**	Promega	*E. coli* cloning vector
**pAcsA-cLac143-YFP**	(Markley et al., 2015)	cLac143 promoter for IPTG inducible expression in *Synechococcus* sp. PCC 7002
**pET28b-crLDHwt**	(Burgess et al., 2016)	pET28b vector for IPTG inducible expression of the *C. reinhardtii* LDH in *E. coli*
**pSJ044**	This work	pUC19 – *acsA*::P_cLac143_-*LDH^WT^*
**pSJ045**	This work	pUC19 – *acsA*::P_cLac143_-*LDH^WT^*+*lldp*
**pSJ041**	This work	pUC19 – *acsA*::P_cLac143_-*LDH^SRT^*
**pSJ042**	This work	pUC19 – *acsA*::P_cLac143_-*LDH^SRT^*+*lldp*
**pSJ046**	This work	pUC19 – *acsA*::P_cLac143_-*LDH^ARSRR^*
**pSJ047**	This work	pUC19 – *acsA*::P_cLac143_-*LDH^ARSRR^*+*lldp*
**pSJ026**	This work	pGEM-T – Syn7002 *flv1* gene with 500 bp up and downstream homology regions
**pSJ028**	This work	pGEM-T – Syn7002 *flv3* gene with 500 bp up and downstream homology regions
**pSJ023**	This work	pUC19 – Syn7002 *pgr5* gene with 500 bp up and downstream homology regions
**pSJ058**	This work	pGEM-T – Syn7002 *flv1*::*CmR*
**pSJ038**	This work	pGEM-T – Syn7002 *flv3*::*SpR*
**pSJ074**	This work	pUC19 – Syn7002 *pgr5*::*EryR*

For D-lactate producing strains, the chloroplast-targeted D-lactate dehydrogenase (LDH) encoded by *Chlamydomonas reinhardtii* (without its cognate transit peptide) was amplified using Q5 polymerase (NEB) from the pET28b-crLDHwt vector (Burgess et al., 2016) and cloned into the pAcsA-cLac143-YFP vector (Markley et al., 2015), substituting the *yfp* gene in its entirety and generating pSJ044. The *lldp* gene (encoding lactate permease) was amplified from *E. coli* Stellar cells and introduced downstream from the *ldh* gene, with the strong AGGAGA RBS (Markley et al., 2015) sequence 8 bp upstream of its start codon, generating pSJ045. To generate a crLDH variant able to utilize NADPH, the sequence of the *C. reinhardtii* LDH was aligned with the sequence of the *Lactobacillus delbrueckii* 11842 LDH (see **Figure S1**) and the nucleotide binding motif (D181 to N185 in the *L. delbrueckii* enzyme) was targeted for site-directed mutagenesis, using previously described variants (Li et al., 2015; Meng et al., 2016) as a guide. The equivalent amino acids in the *C. reinhardtii* LDHwt sequence (D^208^I^209^K^210^P^211^N^212^, see **Figure S1**) were mutated to either S^208^R^209^T^210^ or ARSRR using Restriction-Free cloning (Van Den Ent and Löwe, 2006; Bond and Naus, 2012) in both pSJ044 and pSJ045, resulting in pSJ041 and pSJ042 or pSJ046 and pSJ047, respectively (see **Table 1**).

For KO of the *flv1* (*A1743*), *flv3* (*A1321*) and *pgr5* (*A1477*) genes, DNA sequences from 500 bp up- to 500 bp downstream of the corresponding genes were amplified from Syn7002 genomic DNA and inserted into either pGEM-T Easy (in the case of *flv1* and *flv3*) or an XbaI-digested pUC19 fragment (in the case of *pgr5*), resulting in plasmids pSJ026, pSJ028 and pSJ023, respectively. These plasmids were reverse PCR amplified using the primers stated in **Table S1** and a chloramphenicol-resistance cassette (from pSK9, a kind gift from Annegret Wilde, University of Freiburg), a spectinomycin-resistance cassette (from pBAD42) or an erythromycin-resistance cassette (from pE194, Elhai and Wolk, 1988) were amplified with 15 bp overhangs. These were then assembled into the corresponding linearized plasmid backbones, generating pSJ058, pSJ038, and pSJ074, respectively. These plasmids were then used to transform Syn7002 WT, as described above, alone or in combination, generating the corresponding KO strains. Once full segregation was confirmed (see **Figure S2**), KO strains were transformed with pSJ042, generating D-lactate producing strains (see **Table 2**).

**Table 2:** List of all strains used or generated in this study.

Strain	Genotype
**WT**	*Synechococcus* sp. PCC 7002 wild-type
**cSJ003**	Δ*acs*A::P_cLac143_ *ldh* ^WT^
**cSJ004**	Δ*acs*A::P_cLac143_ *ldh* ^WT^ - *lldP*
**cSJ005**	Δ*acs*A::P_cLac143_ *ldh* ^ARSRR^
**cSJ006**	Δ*acs*A::P_cLac143_ *ldh* ^ARSRR^ - *lldP*
**cSJ007**	Δ*acs*A::P_cLac143_ *ldh* ^SRT^
**cSJ008**	Δ*acs*A::P_cLac143_ *ldh* ^SRT^ - *lldP*
**cSJ030**	Δ*flv1*::CmR
**cSJ019**	Δ*flv3*::SpR
**cSJ111**	Δ*pgr5*::EryR
**cSJ031**	Δ*flv1*::CmR Δ*flv3*::SpR
**cSJ072**	Δ*pgr5*::EryR Δ*flv1*::CmR
**cSJ076**	Δ*pgr5*::EryR Δ*flv3*::SpR
**cSJ042**	Δ*flv1*::CmR Δ*acs*A::P_cLac143_ *ldh* ^SRT^ - *lldP*
**cSJ048**	Δ*flv3*::SpR Δ*acs*A::P_cLac143_ *ldh* ^SRT^ - *lldP*
**cSJ038**	Δ*pgr5*::EryR Δ*acs*A::P_cLac143_ *ldh* ^SRT^ - *lldP*
**cSJ052**	Δ*flv1*::CmR Δ*flv3*::SpR Δ*acs*A::P_cLac143_ *ldh* ^SRT^ - *lldP*
**cSJ075**	Δ*flv1*::CmR Δ*pgr5*::EryR Δ*acs*A::P_cLac143_ *ldh* ^SRT^ - *lldP*
**cSJ079**	Δ*flv3*::SpR Δ*pgr5*::EryR Δ*acs*A::P_cLac143_ *ldh* ^SRT^ - *lldP*

### D-Lactate Concentration Measurements

All D-lactate producing cultures were induced 24 hours after inoculation with the addition of 0.5 mM isopropyl-β-D-thiogalactoside (IPTG, GoldBio) from a sterile 1M stock solution. D-lactate production in cyanobacterial cultures was measured in the culture supernatants, following a centrifugation step (20,000 g, 5 min, room temperature) to remove cells and incubation of the cleared supernatant at 98°C, 5 min, to inactivate remaining enzymatic activity. Cleared and heat-treated supernatants were frozen at -20°C until further use. D-lactate concentrations were measured in duplicate using the D-lactate (Rapid) assay kit (K-DATE, Megazyme) in 96-well plates, following the manufacturer's instructions.

### Western Blotting Analysis

Cultures at the indicated time points were centrifuged (5000 g, 15 min, room temperature), washed once with 1 mM Tris buffer (pH 7.5), and resuspended in the same buffer, supplemented with Protease Inhibitor Tablets, EDTA-free (Pierce). Whole cell lysates were obtained as previously described (Selão et al., 2016), with the exception that cells were lysed using glass beads (Sartorius, 0.17–0.18 mm diameter) and a bead beater (SpeedMill Plus, AnalyticJena). Protein content in cleared whole cell lysates was estimated using the Peterson method (Peterson, 1977) and 15 µg total protein were separated in 12.5% SDS-PAGE precast gels (GE Healthcare). Proteins were transferred to PVDF membranes, probed with specific antibodies raised in rabbit against Flv3 (a kind gift from Toshiharu Shikanai, Kyoto University; see Yamamoto et al., 2016), secondary goat anti-rabbit HRP-conjugated antibodies, and developed as described (Selão et al., 2016).

## Results

### Construction of D-Lactate Producing Strain of PCC 7002

**Figure 2:**
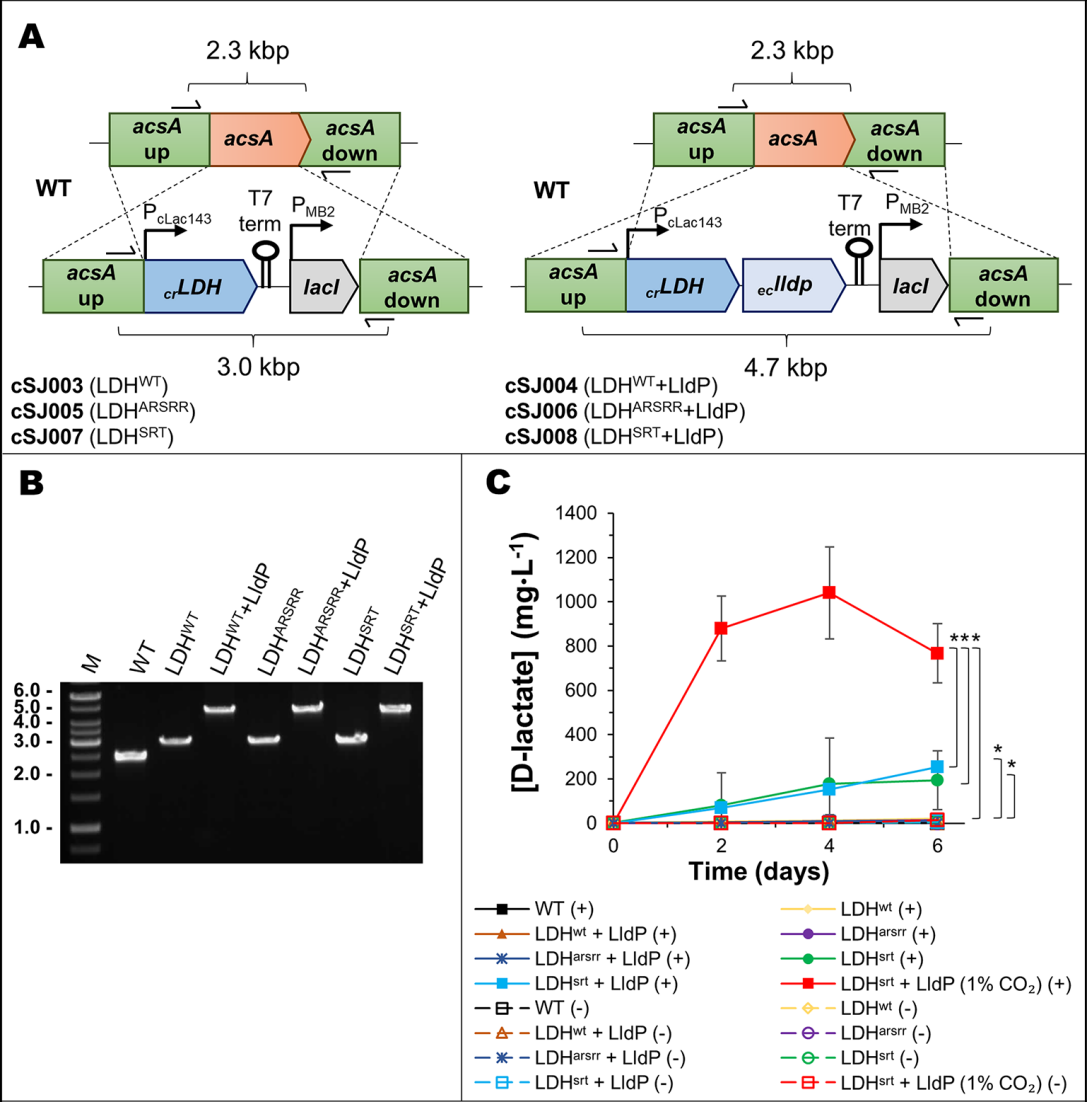
Cloning and production test of a mutated D-lactate dehydrogenase in Syn7002. **(A)** Schematic of the *acsA* gene locus modification in strains cSJ003/cSJ005/cSJ007 (*left*, with *ldh* gene only) and strains cSJ004/cSJ006/cSJ008 (*right*, with *ldh* and *lldp* genes in operon). Different components not to scale. **(B)** 1% agarose gel of colony PCR of the strains indicated, using primers shown in A). **(C)** D-lactate production test of the strains indicated, at 38°C, in either ambient air or 1% CO_2_, and a light intensity of 250 µmol photons·m^-2^·s^-1^. Error bars indicate a 95% confidence interval based on the standard error of the mean for triplicate cultures. Asterisks denote statistically relevant differences between indicated strains.

To test the impact of re-routing photosynthetic electron flow on biosynthesis, we generated a strain of the cyanobacterium Syn7002 that produces D-lactate, which can be easily assayed and is an important chemical feedstock in its own right (Eiteman and Ramalingam, 2015). We introduced into Syn7002 a DNA sequence encoding the recently characterized NAD^+^-specific D-LDH (D-nLDH; EC 1.1.1.28) from the green alga *Chlamydomonas reinhardtii* (Burgess et al., 2016) and generated two derivatives (LDH^SRT^ and LDH^ARSRR^) that were re-engineered on the basis of previous work to change the co-enzyme specificity of the enzyme to allow the use of NADPH (Li et al., 2015; Meng et al., 2016). All stains were constructed so that transcription of the D-LDH genes was under the control of the cLac_143_ promoter (thus inducible by IPTG) and included downstream the *E. coli lldP* gene encoding a lactate permease, previously shown to improve D-lactate export in cyanobacteria (Li et al., 2015) (**Figure 2**). Analysis of D-lactate production under photoautotrophic conditions (0.04% or 1% CO_2_ (v/v) and 38°C) revealed that the highest titer (≈1 g/L, 4 days post-induction, at 38°C and 1% (v/v) CO_2_) occurred with the LDH^SRT^ strain co-expressing LldP (cSJ008, **Figure 2**), which was therefore used as the test or control strain for all subsequent experiments. All other strains produced less than 20 mg/L.

### Effect of Single Knock-Outs of the *flv1*, *flv3,* and *pgr5* Genes in Different Conditions

**Figure 3:**
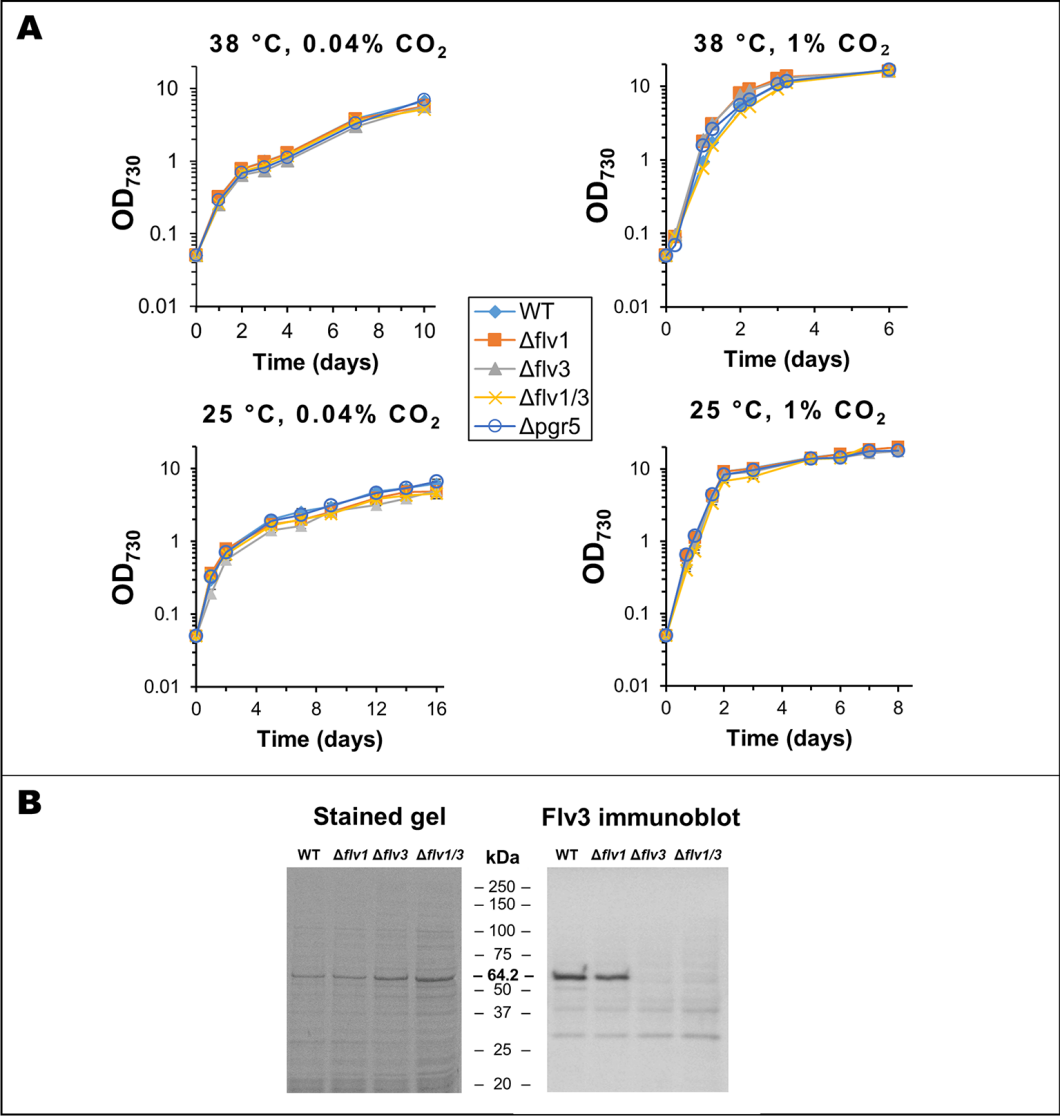
Analysis of *flv* and *pgr5* knock-out strains. **(A)** Growth curves for WT, *Δflv1*, *Δflv3*, *Δflv1*/*Δflv3*, and *Δpgr5* strains in different conditions. Data is an average of 3 biological replicates, measured in technical duplicates. In all cases, the total light intensity was 250 µmol photons·m-2·s-1. **(B)** Analysis of Flv3 expression in different strains. *Left panel*: Coomassie stained SDS-PAGE gel of whole cell lysates from WT and different flv deletion strains. *Right panel*: Immunoblot using same samples, developed using a specific anti-Flv3 antibody (a kind gift from Toshiharu Shikanai). All lanes loaded with 15 µg total protein. Numbers on the left of each image represent the migration of respective molecular weight markers.

In Syn6803, there are 4 Flv proteins (Flv1-4), which form two distinct heterodimeric complexes (Flv1/3 and Flv2/4) with roles in oxygen reduction downstream of PSI (Zhang et al., 2012; Allahverdiyeva et al., 2013; Shimakawa et al., 2015; Santana-Sanchez et al., 2019). In addition, it remains possible that under certain conditions, all four Flv proteins might form homodimers of unknown function (Hackenberg et al., 2009; Mustila et al., 2016; Santana-Sanchez et al., 2019). Only two Flv proteins, annotated as Flv1 and Flv3, based on similarities to the Flv subunits in Syn6803, are found in Syn7002. However, detailed studies on the role of Flv1 and Flv3 in Syn7002 are lacking, with only a double *flv1/flv3* KO mutant so far studied in detail (Shimakawa et al., 2016). In order to ascertain the baseline effect of the different mutations, growth of the strains with single gene deletions in either *flv1* (cSJ030), *flv3* (cSJ019), or *pgr5* (cSJ111) was monitored under different growth conditions (**Figure 3A**). Knock-out (KO) of either of the *flv* genes in Syn7002 resulted in only a mild growth defect at 25°C and at ambient CO_2_ concentrations (final cell density between 73% and 80% of WT cultures under the same conditions). However, either increased temperature or higher CO_2_ concentration (even at 25°C) allowed the cells to grow at near WT rates (**Figure 3A**) and accumulate similar cell densities. An immunoblot analysis (**Figure 3B**) using a specific antibody for Flv3 showed that Flv3 still accumulated upon deletion of the *flv1* gene, supporting the possibility that Flv3 is capable of functioning independently of Flv1 in Syn7002. The Δ*pgr5* strain, on the other hand, showed no obvious growth defect in all conditions tested (**Figure 3A**). Attempts to inactivate the NDH pathway by deleting *ndhF1* proved to be impractical as the resulting strain grew extremely poorly under the growth conditions used (data not shown).

**Figure 4:**
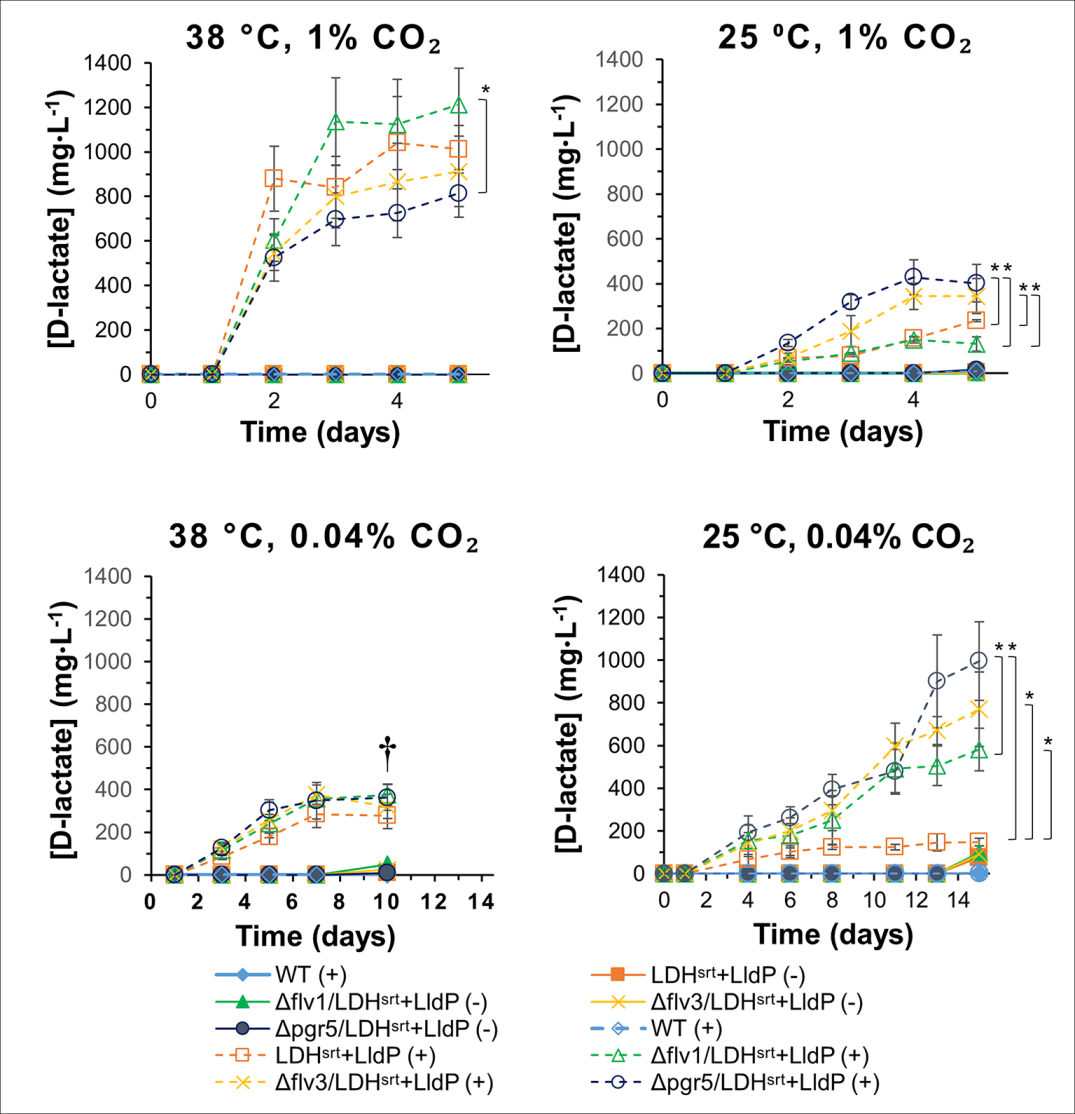
D-lactate production in several electron transport single mutants under different growth conditions. D-lactate concentrations in culture supernatants were measured at the time points indicated, in the absence (-) or presence (+) of 0.5 mM IPTG. Error bars indicate a 95% confidence interval based on the standard error of the mean for triplicate cultures. Asterisks denote statistically relevant differences between indicated strains. † - Culture death.

Expression of the LDH^SRT^-LldP operon in the single KO strains had very different effects depending on growth conditions. At 38°C and high CO_2_ conditions [1% (v/v)], all strains were able to produce D-lactate efficiently (**Figure 4**), with the producing strain in the *flv1* KO background (cSJ042) excreting D-lactate at slightly greater levels than that of the WT control strain (cSJ008), although the difference was not statistically significant. In contrast, deletion of *pgr5* (cSJ038) negatively affected D-lactate production under this growth regime. At 25°C and high CO_2_, however, this effect was reversed, with strain cSJ038 (lacking *pgr5*) producing significantly more D-lactate than the WT control strain. Strain cSJ048 (lacking *flv3*) also produced significantly more D-lactate than strain cSJ042 (lacking *flv1)* (**Figure 4**), again suggesting that the two *flv* paralogs are not equivalent in Syn7002. Curiously, the growth of strains cSJ038 and cSJ048 was more severely affected than that of strains cSJ008 and cSJ042 (**Figure S3**).

Under air CO_2_ concentrations, all single KO strains displayed slightly higher D-lactate production titers at 38°C than the control strain, cSJ008, reaching ≈350-370 mg/L vs ≈280 mg/L, respectively (**Figure 4**); though, again, deletion of *flv3* or *pgr5* affected growth rates more severely than deletion of *flv1* (**Figures S3** and **S4**). At 25°C and ambient CO_2_, the effect of the different mutations was more obvious, with the strains producing approximately 4 to 6.5-fold more D-lactate than the WT control strain (cSJ008) (≈580 to ≈1000 mg/L compared to ≈150mg/L, **Figure 4**). Strain cSJ038 (lacking *pgr5*) gave the highest titer and was able to produce ≈1 g/L of D-lactate at the endpoint of the experiment (**Figure 4**). Under these growth conditions, the *flv* KO cultures were severely affected, having lower chlorophyll content than both cSJ008 and cSJ038 at 5 days post-induction (**Figures S5A, B**), though cell density was on par with the remaining strains (**Figure S3**).

### Effect of Double Knock-Outs of the *flv1*, *flv3,* and *pgr5* Genes in Different Conditions

**Figure 5:**
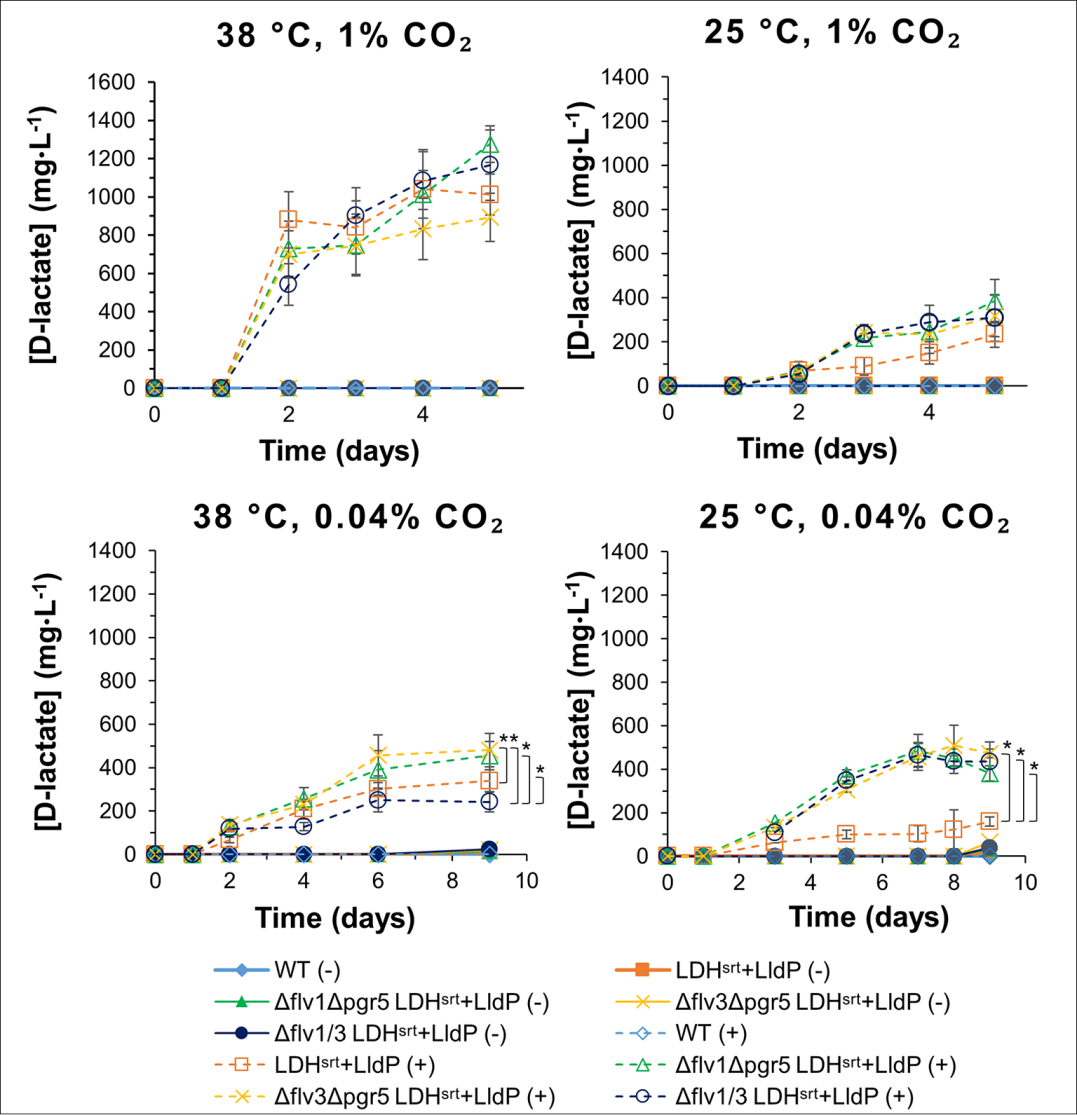
D-lactate production in several electron transport double mutants under different growth conditions. D-lactate concentrations in culture supernatants were measured at the time points indicated, in the absence (-) or presence (+) of 0.5 mM IPTG. Error bars indicate a 95% confidence interval based on the standard error of the mean for triplicate cultures. Asterisks denote statistically relevant differences between indicated strains.

Given that the *flv1* and *flv3* single KO mutants behaved differently, we set out to compare D-lactate production in double *flv1*/*flv3* KO strains as well as in *flv* KO combinations with Δ*pgr5*, in different growth conditions. At 38°C and 1% (v/v) CO_2_, the effect of different double KO combinations was minimal, with a very marginal decrease in production titers in the Δ*flv3*/Δ*pgr5* (cSJ079) strain (**Figure 5**); whereas at 25°C and 1% (v/v) CO_2_, all KO combinations had a clear positive effect on D-lactate production, resulting in increased productivity per cell density (**Figure S7**), though at the cost of stunted growth and decreased strain fitness (**Figure S6**). Under ambient CO_2_ conditions, the effect on D-lactate production of the different background mutations was highly dependent on temperature. While at 38°C, combinations of *flv1* or *flv3* KO with *pgr5* KO increased D-lactate production, the double *flv1*/*flv3* KO had a slightly negative impact, both in terms of titer (**Figure 5**) as well as in per cell density productivity (**Figure S7**). As mentioned, under these conditions, single *flv* gene deletions (in cSJ042 and cSJ048, **Figure 4**) did not decrease D-lactate production, which again supports the idea that Flv1 and Flv3 may be able to act independently to some extent.

At lower temperature, all strains behaved similarly, with a strong enhancement of D-lactate production titers (**Figure 5**) and per cell density productivities (**Figure S7**), even though growth was affected in all double KO strains (**Figure S6**). It should be noted that the double KO backgrounds did not allow as high production titers at low temperature and ambient CO_2_ concentrations as the single KO strains (**Figure 4**), with double KO strains having a stunted growth in comparison to single KO strains (**Figure S6**). In that respect, the enhancement of D-lactate production granted by the single *pgr5* KO at 25°C and ambient CO_2_ seems to be negated in the double KO strains when any of the *flv* genes was also deleted, though, interestingly, chlorophyll content of the D-lactate producing double KO strains was not as severely affected in comparison to strain cSJ008 as that of single KO strains (**Figure S5** and **S8**), though this may also be related to the overall lower production titers attained by the double KO strains.

### pH Control Improves D-Lactate Productivities

Given that D-lactate production at g/L concentrations severely decreases the pH of the medium to an average pH of between 5-6 four days post-induction (data not shown), thus inhibiting culture growth, different strategies were devised to test whether pH control would result in increased production. As shown in **Figure S9**, addition of extra buffer (50 mM HEPES, pH 8.0) allowed an increase in production titers of roughly 30% after 3 days. However, cultures suffered a significant decline after the third day, with D-lactate concentrations also dropping. On the other hand, adjusting pH to ≈8.2 by adding a strong inorganic base (NaOH) whenever culture pH dropped below 7 had a better effect, with production after 3 days reaching ≈1.45 g/L, a titer almost double that of the previously reported maximum for D-lactate producing strains of Syn7002 grown under similar conditions (Gordon et al., 2016). It is likely that using a continuous pH–monitoring online system would help to further improve production.

## Discussion

Our work has demonstrated that the manipulation of alternative electron transfer pathways associated with photosynthetic electron flow is a valid strategy for increasing photosynthetic production of biomolecules from the pyruvate node without utilizing a CO_2_-enriched air supply.

Our results support the hypothesis, based on previous work in Syn6803 (Helman et al., 2003) and Syn7002 (Shimakawa et al., 2016), that loss of the Flv1 and Flv3 subunits in Syn7002 leading to over-reduction of the acceptor side of PSI in the light, thereby likely increasing the ratio of [NADPH]/[NADP^+^] in the cell, improves the rate of reduction of pyruvate to D-lactate by an NADP*^+^*-dependent LDH. Previous work has shown that *flv* transcript levels increase in Syn7002 in response to low CO_2_ concentrations but not in response to a transient high light treatment (Ludwig and Bryant, 2012a). These observations correlate well with the observed increase in D-lactate production in Δ*flv* strains at low temperature and ambient CO_2_ concentrations — conditions that promote elevated levels of NADPH due to slowed CO_2_ fixation and reduced consumption of NADPH and ATP in the Calvin-Benson cycle. Furthermore, transcript levels for both *flv* genes were shown to increase in Syn7002 in response to low temperature, signaling that these genes may be important in the response to low temperature stress (Ludwig and Bryant, 2012b) and that their deletion under these conditions may therefore lead to a more severe response than at standard (higher temperature) conditions. Recent work also suggests that deletion of *flv* genes has pleiotropic effects in Syn6803 (Mustila et al., 2016), which might contribute to the growth defects we see in the various single and double *flv* mutants.

Previous studies on the two Flv subunits in Syn7002 has been limited to the analysis of a double KO mutant (Shimakawa et al., 2016). Our results suggest that disruption of either of the two *flv* homologues can disrupt the redox balance in the cell by (perhaps partially) disrupting alternative electron flow and PSI oxidation, especially at lower temperatures and CO_2_ concentrations. However, at least in some conditions (such as at lower temperature and high CO_2_), Flv1 and Flv3 are not functionally equivalent, with Flv3 playing a more important physiological role, in line with previous studies in Syn6803 (Hackenberg et al., 2009; Mustila et al., 2016). The heterodimeric complex of Flv1/3 was previously shown to have an important role in the rapid response of Syn6803 to fluctuating light (Allahverdiyeva et al., 2013), with the Flv2/4 heterodimer contributing mostly to a steady-state response induced by low carbon availability (Shimakawa et al., 2015; Santana-Sanchez et al., 2019). It is therefore possible that growing *flv* mutants under fluctuating light might further enhance D-lactate production through transient perturbations in the ratio of [NADPH]/[NADP^+^]. Even though the transcriptome of *flv* mutant strains of Syn6803 has been thoroughly characterized in previous studies (Mustila et al., 2016), so far there has been no attempt to study changes to the global metabolome in response to these mutations. Given that Flv proteins are currently considered to be part of an electron overflow pathway to oxygen, our results could be explained by an increase in the intracellular [NADPH]/[NADP^+^] ratio. However, in view of the pleiotropic effects of these mutations observed in previous studies (Mustila et al., 2016), a complete metabolome study will be necessary in order to more fully understand the full impact of *flv* and *pgr5* deletions in different cyanobacteria.

Disruption of the *pgr5* gene had a surprisingly strong effect on D-lactate production at lower temperatures and CO_2_ concentrations. While little is known about the function of this protein in cyanobacteria, our results suggest that it has an important role in alternative electron flow and redox balance in the cell under limiting CO_2_ and/or lower temperature. Strikingly, a D-lactate producing strain with a *pgr5* deletion, while only able to accumulate to lower cell densities, had higher per cell density productivity (**Figure S4**) and almost no loss of chlorophyll content in comparison to induced cSJ008 (**Figure S5**). Previously, an *A. thaliana pgr5-*null mutant was shown to have a significant growth defect at high light intensities (>150 µmol photons·m^-2^·s^-1^) when grown in ambient CO_2_ conditions, a defect that could be partially suppressed when the strain was grown under CO_2_-enriched air (Munekage et al., 2008). This was argued to be due to a shortfall in ATP synthesis that hindered effective carbon fixation in low CO_2_ conditions combined with enhanced photoinhibition of PSI owing to an over-reduced acceptor side. Though further studies will be required to accurately pinpoint the mechanism of action of *pgr5* in Syn7002, deletion of *pgr5* may be a valid strategy to enhance production of molecules from the pyruvate node at ambient temperature (thus obviating the extra energy expenditure to keep cultures at the relatively high temperature of 38°C) and CO_2_ concentrations. While the strains developed here do require longer timespans to reach similar titers to WT background strains grown at elevated temperature and CO_2_ concentrations, our work demonstrates that high titers are achievable under “standard” conditions, with further developments now focusing on decreasing the production time so as to increase economic viability of the process.

As recently discussed by Erdrich and co-workers (Erdrich et al., 2014), there is a discrepancy between the required ATP/NADPH ratio for biomass production (1.51), the ratio derived from photosynthetic linear electron flow (1.28) and that required for, e.g., efficient ethanol production (1.17). Therefore, in order to efficiently redirect carbon flux from biomass to product formation, mutations decreasing the ATP/NADPH ratio (by either increasing NADPH concentration and/or increasing ATP turnover rate) can have beneficial effects on production. One of the suggested modifications to achieve this goal is the disruption of the pseudocyclic electron flow catalyzed by the Flv proteins, a suggestion that is supported by the findings in this work. Overall, our study validates the concept that re-routing cyclic and pseudocyclic electron flow in cyanobacteria is a viable strategy to improve direct bioconversion of CO_2_ to relevant products (such as D-lactate) in a sustainable, eco-friendly manner.

## Data Availability Statement

All datasets generated for this study are included in the article/**Supplementary Material**.

## Author Contributions

TS, BN and PN devised the experiments. TS, NI and JJ performed the experiments. TS, JJ, BN and PN analyzed the data. TS and PN wrote the manuscript.

## Funding

This work was supported by NTU grants M4080306 to BN and M4081714 to PN.

## Conflict of Interest

The authors declare that the research was conducted in the absence of any commercial or financial relationships that could be construed as a potential conflict of interest.
