# Supplementary material for: Enhanced Production of D-Lactate in Cyanobacteria by Re-Routing Photosynthetic Cyclic and Pseudo-Cyclic Electron Flow
Source: Front Plant Sci. 2020 Jan 31;10:1700. doi: 10.3389/fpls.2019.01700 (PMC7025493; doi:10.3389/fpls.2019.01700)
Supplement: Supplementary file 1 [file DataSheet_1.pdf]

Primers used in this study

Name	Sequence
pGEMT_flv1_F	TGGCGATCGCCTTAAGCAGG
pGEMT_flv1_R	TTAGTAAAGGGTCGCAGCAGTTGC
pGEMT_flv3_F	GGGTGTTGTGCCAAACTGCTG
pGEMT_flv3_R	AATACTCAAGCTATGCATCCAACG
InF_pgr5_F	ACCCGGGGATCCTCT ATGGCTCAGATTATTGTCGGTTTG
InF_pgr5_R	CTGCAGGTCGACTCT TGAAGGCTGTCAGAAGTCCTG
InF_UCH_EryR_F	ATTAAGGAGTGGACA TCGCATCCGATTGCAGTAT
InF_UCH_EryR_R	ACGTGAACCAAGTCA ATACTGCAATCGGATGCGA
InF_UCH_CmR_F	ATTAAGGAGTGGACA TTATGCCATGGAGAGTAAAATCC
InF_UCH_CmR_R	ACGTGAACCAAGTCA AGTAAAGCAGTCATAAAGTCTTTTTTATTACG
InF_UCH_SpR_F	ATTAAGGAGTGGACA ACTGGAAACGGATGAAGG
InF_UCH_SpR_R	ACGTGAACCAAGTCA TGCTTTTTAAGGGAATTGTGC
InF_UCH_pUC_flv1_F2	TGACTTGGTTCACGT ATCGCCTTTTAGAATAGGGTTC
InF_UCH_pUC_flv1_R2	TGTCCACTCCTTAAT TAAAATTTATCGCCCCAATTGG
InF_UCH_pUC_flv3_F2	TGACTTGGTTCACGT ACTTGAATTTTTAGCTTTCACAGAG
InF_UCH_pUC_flv3_R2	TGTCCACTCCTTAAT TAACCCTCCTACACTGTG
InF_UCH_pUC_pgr5_F2	TGACTTGGTTCACGT TTGCATTCTTAGGTACTCACTATG
InF_UCH_pUC_pgr5_R2	TGTCCACTCCTTAAT TGCAACTAGCCTCGTAAAAC
pgr5_seg_F	AATTCGGTCAACTCAACAGCTTTG
pgr5_seg_R	AACCCTAAGATCTTCATCCCTAAGG
flv1_seg_F	AGGGATTAGGGCATTGAGTTTGC
flv1_seg_R	TCCAGGGACGGTCAAATTCTC
flv3_seg_F	AACCCCTAATAAATTCGTGCCAC
flv3_seg_R	TCGCCTGCAATGTTTACTGTTTTATTTTAATC
RF_LDH_ARSRR_F	GTATCGGTTGCAAAGTTTTCGCGTACGCCCGTTCCCGTCGC
RF_LDH_ARSRR_R	CCATCGCTTCAACTGCCGGGCGACGGGAACGGGC
RF_LDH_SRT_F	GGTATCGGTTGCAAAGTTTCGCGTACTCCCGTACCCCGAACCCGGCAGTTGAAGC
RF_LDH_SRT_R	GCTTCAACTGCCGGGTTCGGGGTACGGGAGTACGCGAAAACTTTGCAACCGATACC
pAcsA_bef_pMB2_F	ATCCGGCTGTCTAACAAAG
pAcsA_lacO_R	GGAATTAATCTCCTACTTGACTTTATG
InF_LDH_to_acsA_F	taggagattaattcc ATGGCGCTGAACGTTGG
InF_LDH_to_acsA_R	gttagacagccggat TCAGTGGTGGTGGTGGTG
InF_LDH_for_LldP_R	TGTCTCCTAAATAGT TCAGTGGTGGTGGTGGTG
InF_LldP_for LDH_F	ACTATTTAGGAGACACATACCATGAATCTCTGGCAACAAAACTACG
InF_LldP_to_acsA_R	gttagacagccggat TTAAGGAATCATCCACGTTAAGAC
