# Supplementary material for: Enhanced Production of D-Lactate in Cyanobacteria by Re-Routing Photosynthetic Cyclic and Pseudo-Cyclic Electron Flow
Source: Front Plant Sci. 2020 Jan 31;10:1700. doi: 10.3389/fpls.2019.01700 (PMC7025493; doi:10.3389/fpls.2019.01700)
Supplement: Supplementary file 2 [file DataSheet_2.pdf]

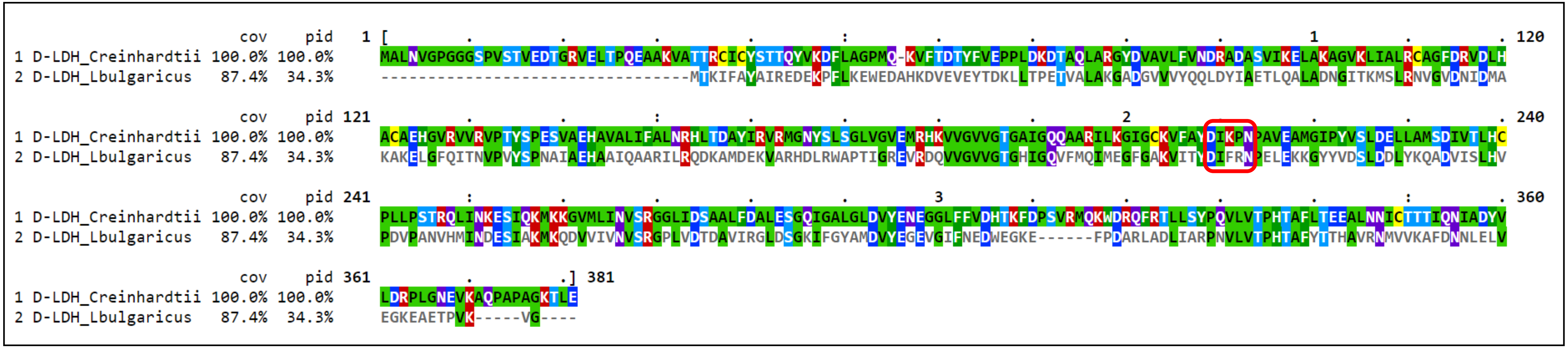

Figure S1 – Sequence alignment of the (truncated) *C. reinhardtii* and the *L. delbrueckii* subsp. *bulgaricus* ATCC 11842 D-LDH enzymes. Alignment was generated with ClustalOmega and visualized using MView. The red outline box indicates the nucleotide discrimination region targeted in this study.

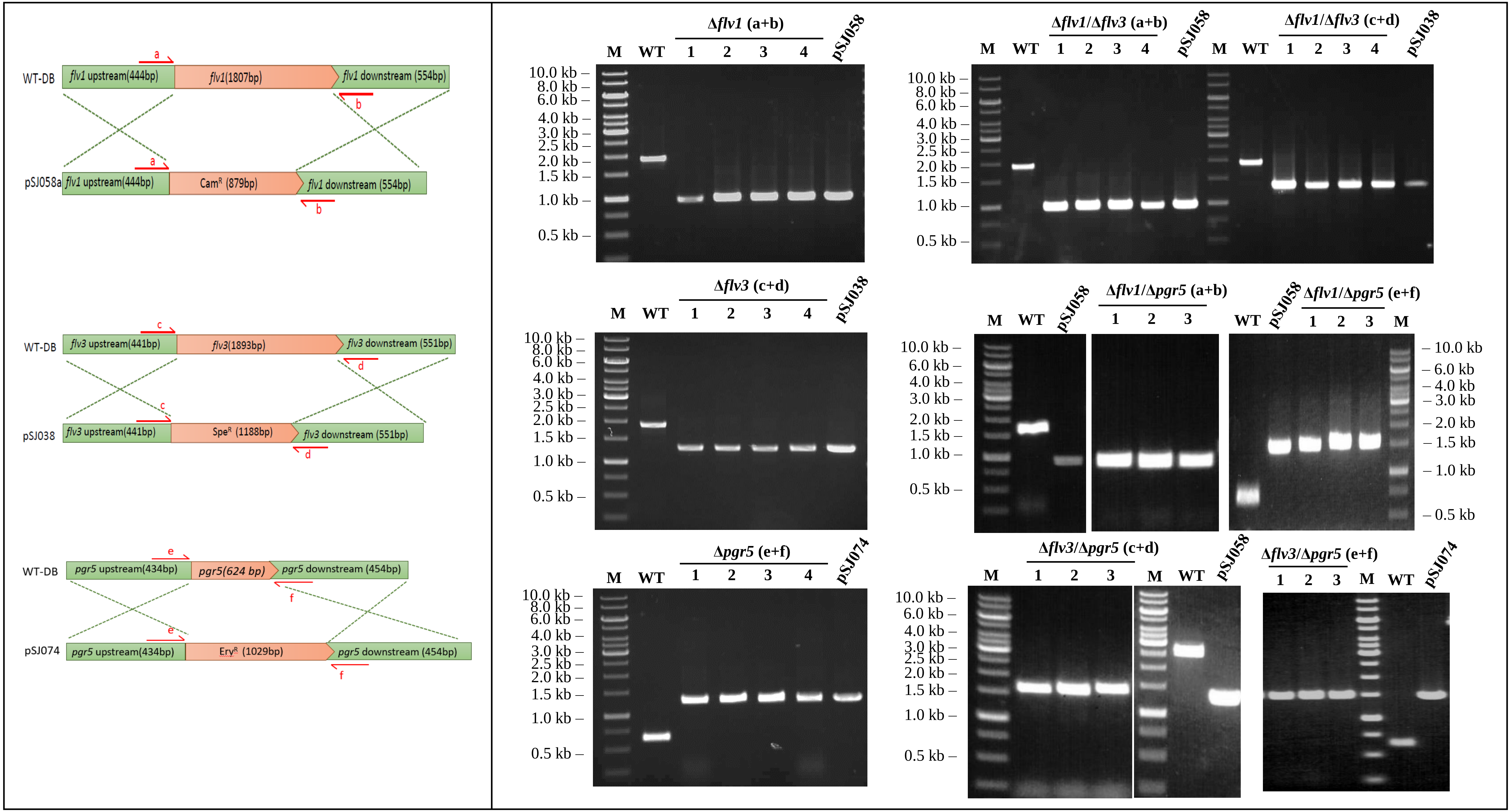

Figure S2 – Schematic of constructs for (*left panel*) and genomic DNA PCR analysis of (*right panel*) corresponding *flv1*, *flv3* and *pgr5* deletion strains.

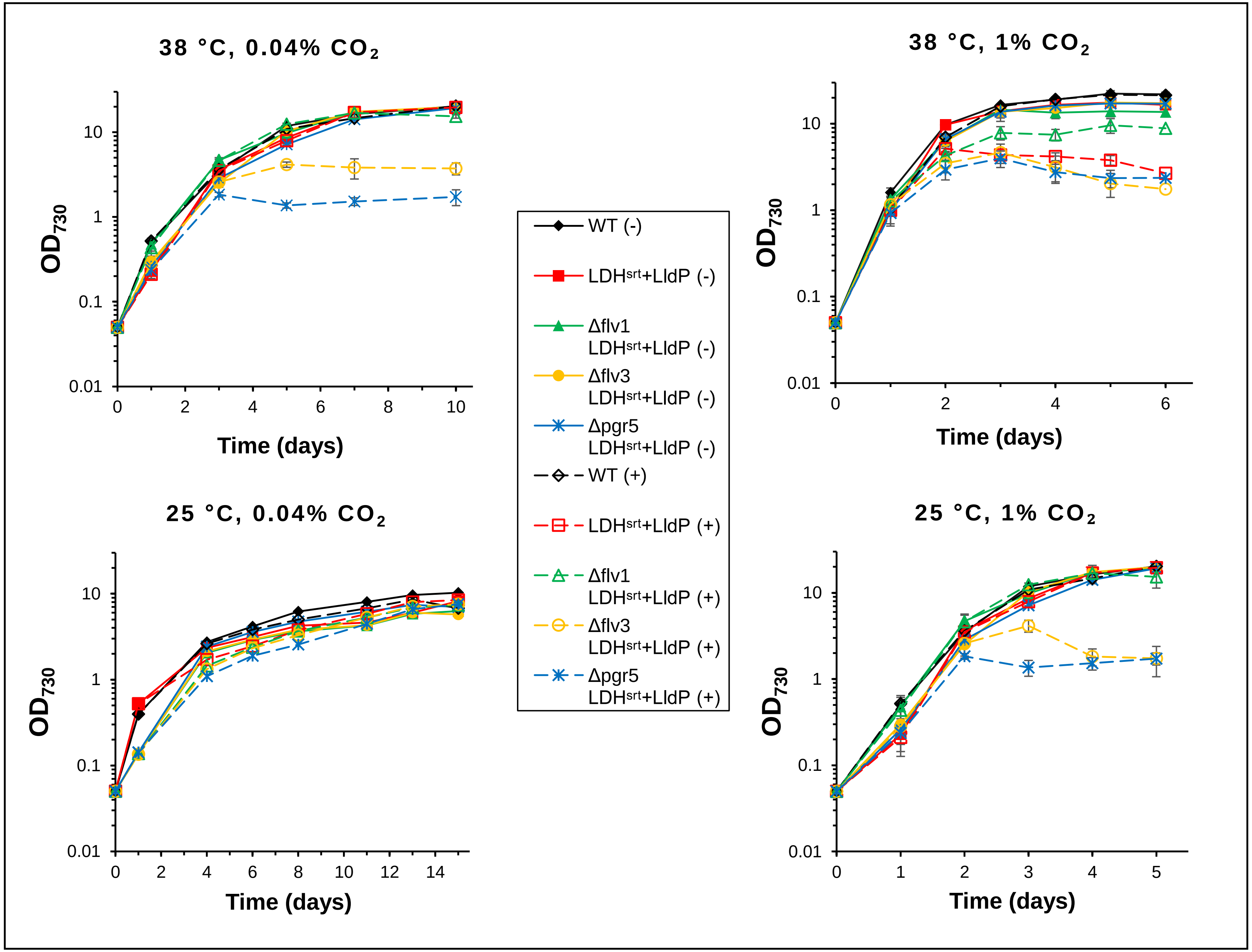

Figure S3

Growth curves for PCC 7002 WT, cSJ008 (LDHsrt_lldP), cSJ042 (Δ*flv1*+LDHsrt_lldP), cSJ048 (Δ*flv3*+LDHsrt_lldP) and cSJ038 (Δ*pgr5*+LDHsrt_lldP) strains in the absence (-) or presence (+) of 0.5 mM IPTG, in different conditions. Data is an average of 3 biological replicates, measured in technical duplicates. Error bars represent standard deviation (n=3). In all cases the total light intensity was 250 µmol photons·m^{-2}·s^{-1}.

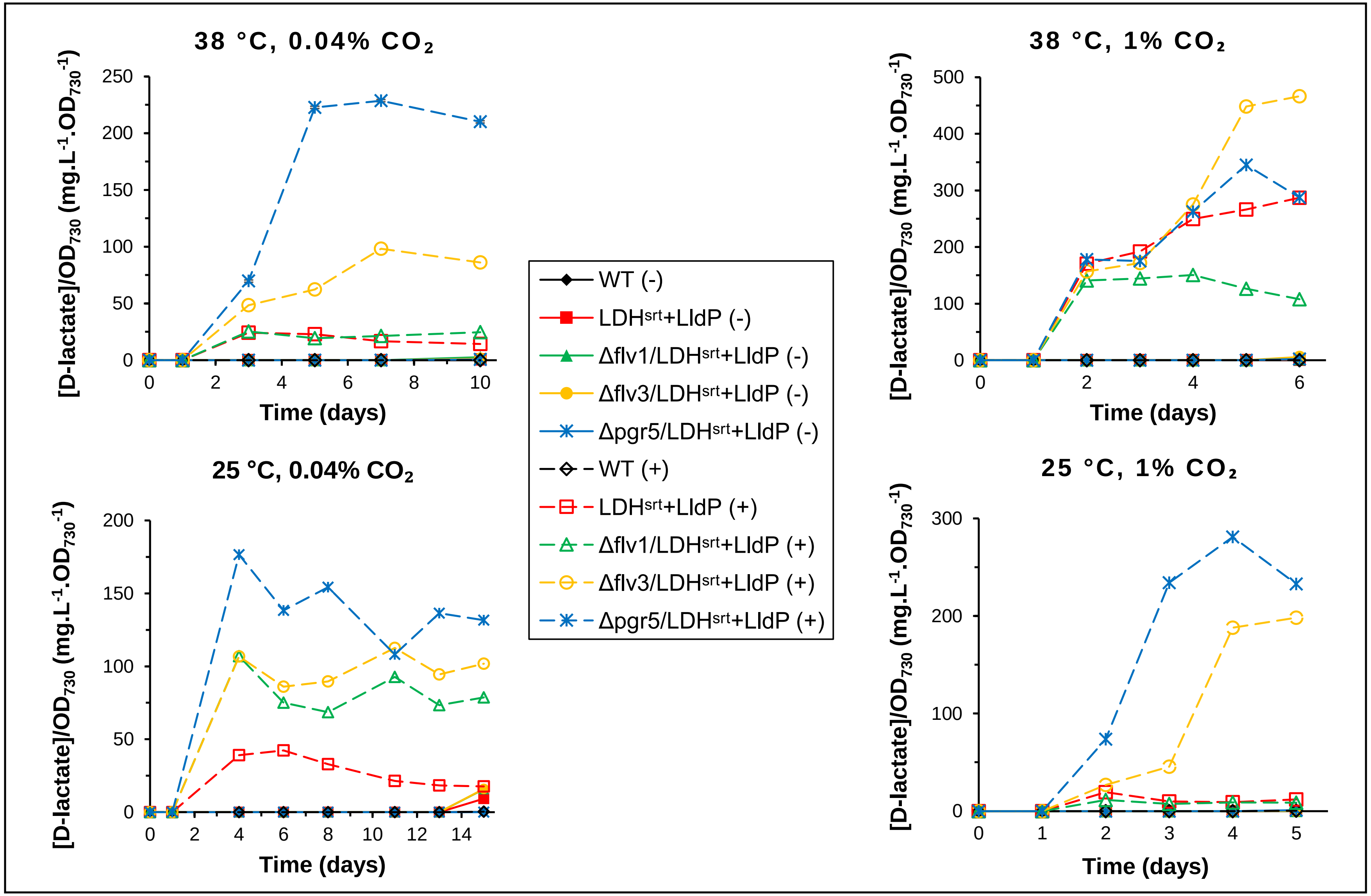

Figure S4

D-lactate production per OD_{730} for PCC 7002 WT, cSJ008 (LDHsrt_lldP), cSJ042 (Δ*flv1*+LDHsrt_lldP), cSJ048 (Δ*flv3*+LDHsrt_lldP) and cSJ038 (Δ*pgr5*+LDHsrt_lldP) strains in the absence (-) or presence (+) of 0.5 mM IPTG, in different conditions. Data is an average of 3 biological replicates, measured in technical duplicates. In all cases the total light intensity was 250 µmol photons·m^{-2}·s^{-1}.

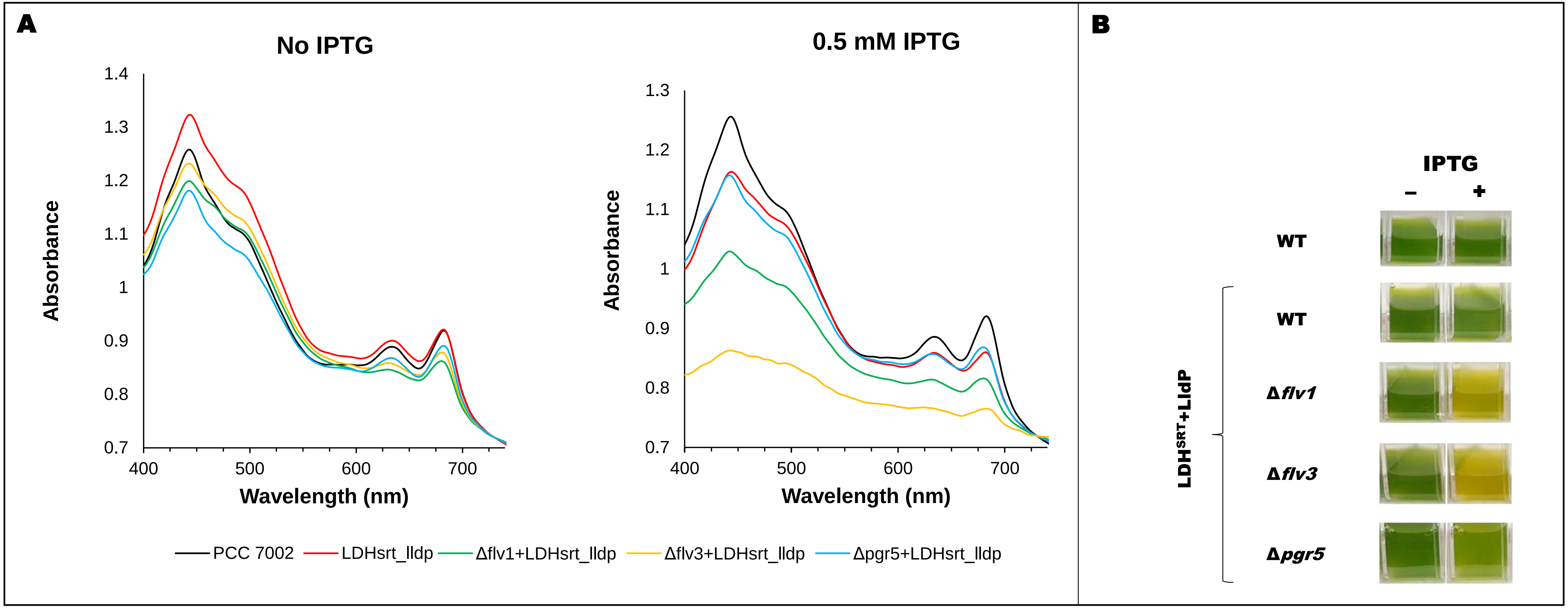

Figure S5

Pigmentation analysis of different D-lactate producing strains (WT background vs single knockout strains).

A) Whole cell absorbance spectra for PCC 7002 WT, cSJ008 (LDHsrt_lldP), cSJ042 (Δ*flv1*+LDHsrt_lldP), cSJ048 (Δ*flv3*+LDHsrt_lldP) and cSJ038 (Δ*pgr5*+LDHsrt_lldP) strains after 5 days at 25 °C and 0.04% CO_2, in the absence (left panel) or presence (right panel) of 0.5 mM IPTG. All spectra were normalized relative to their absorbance at 730 nm.

B) Representative images of the same cultures. Pictures taken at the same time point.

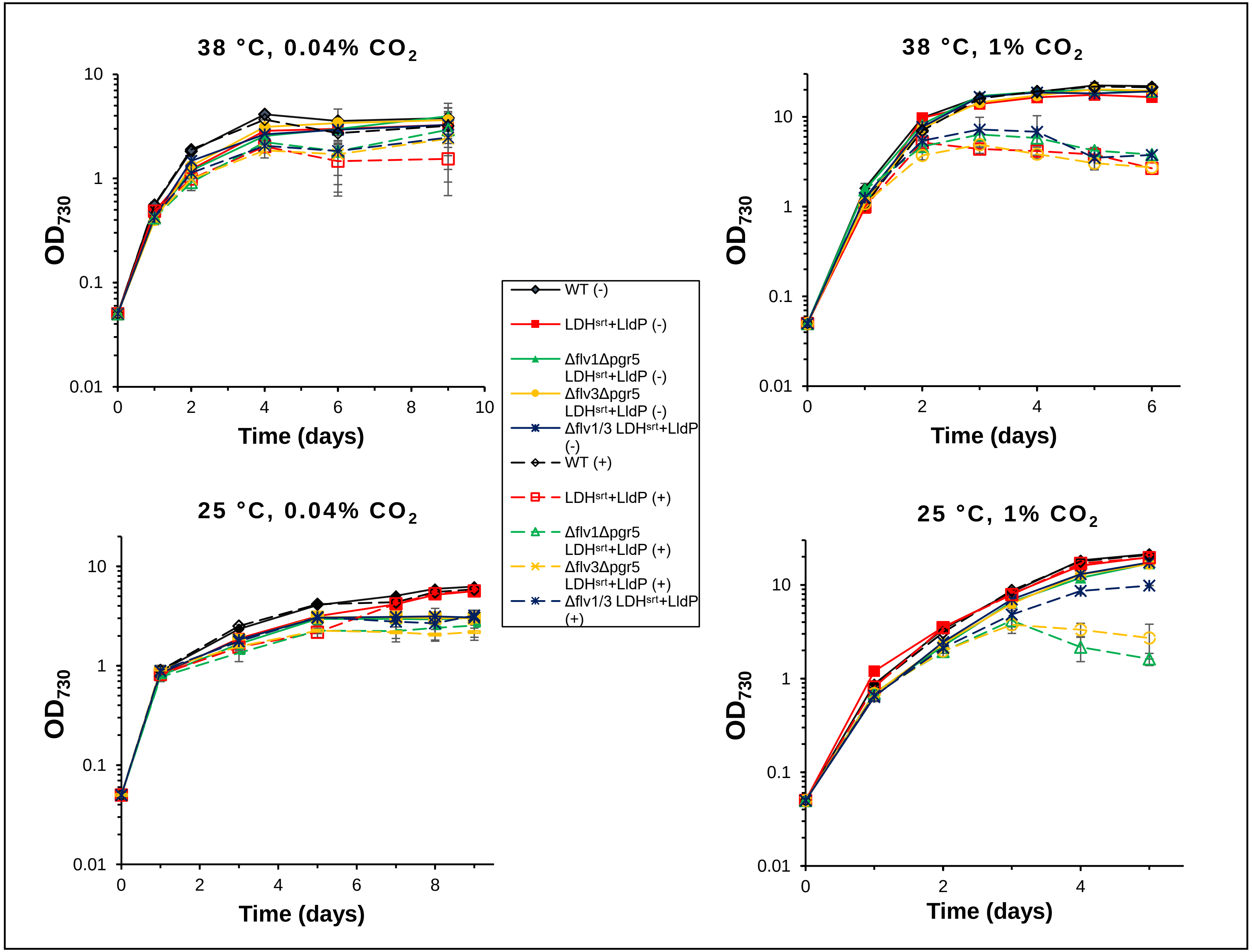

Figure S6
Growth curves for PCC 7002 WT, cSJ008 (LDHsrt_lldP), cSJ075 (*Δflv1/Δpgr5*+LDHsrt_lldP), cSJ079 (*Δflv3/Δpgr5*+LDHsrt_lldP) and cSJ052 (*Δflv1/Δflv3* +LDHsrt_lldP) strains in the absence (-) or presence (+) of 0.5 mM IPTG, in different conditions. Data is an average of 3 biological replicates, measured in technical duplicates. Error bars represent standard deviation (n=3). In all cases the total light intensity was 250 µmol photons$\cdot m^{-2}\cdot s^{-1}$.

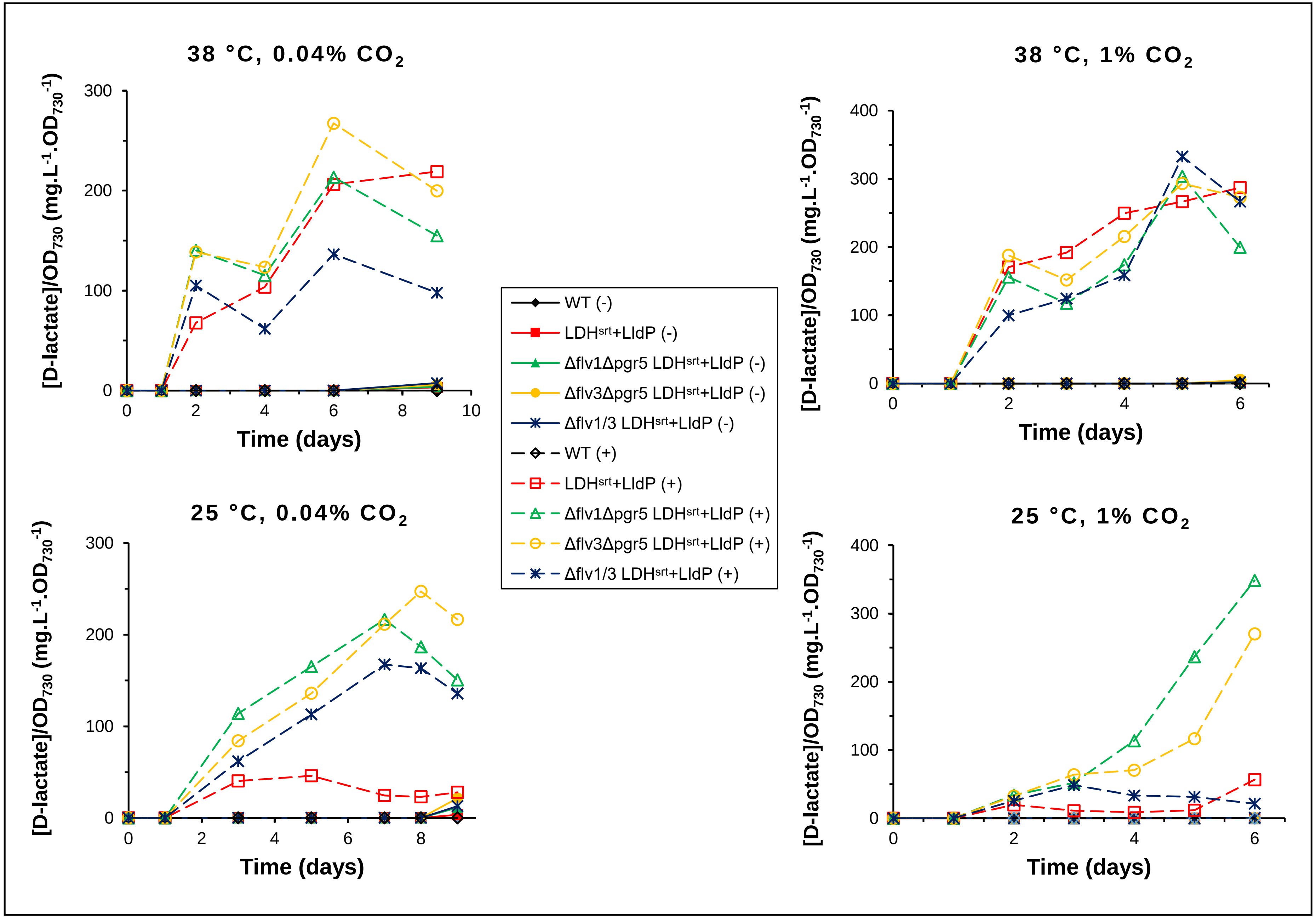

Figure S7

D-lactate production per OD_{730} for PCC 7002 WT, cSJ008 (LDHsrt_lldP), cSJ075 (Δ*flv1*/Δ*pgr5*+LDHsrt_lldP), cSJ079 (Δ*flv3*/Δ*pgr5*+LDHsrt_lldP) and cSJ052 (Δ*flv1*/Δ*flv3* +LDHsrt_lldP) strains in the absence (-) or presence (+) of 0.5 mM IPTG, in different conditions. Data is an average of 3 biological replicates, measured in technical duplicates. In all cases the total light intensity was 250 μmol photons·m^{-2}·s^{-1}.

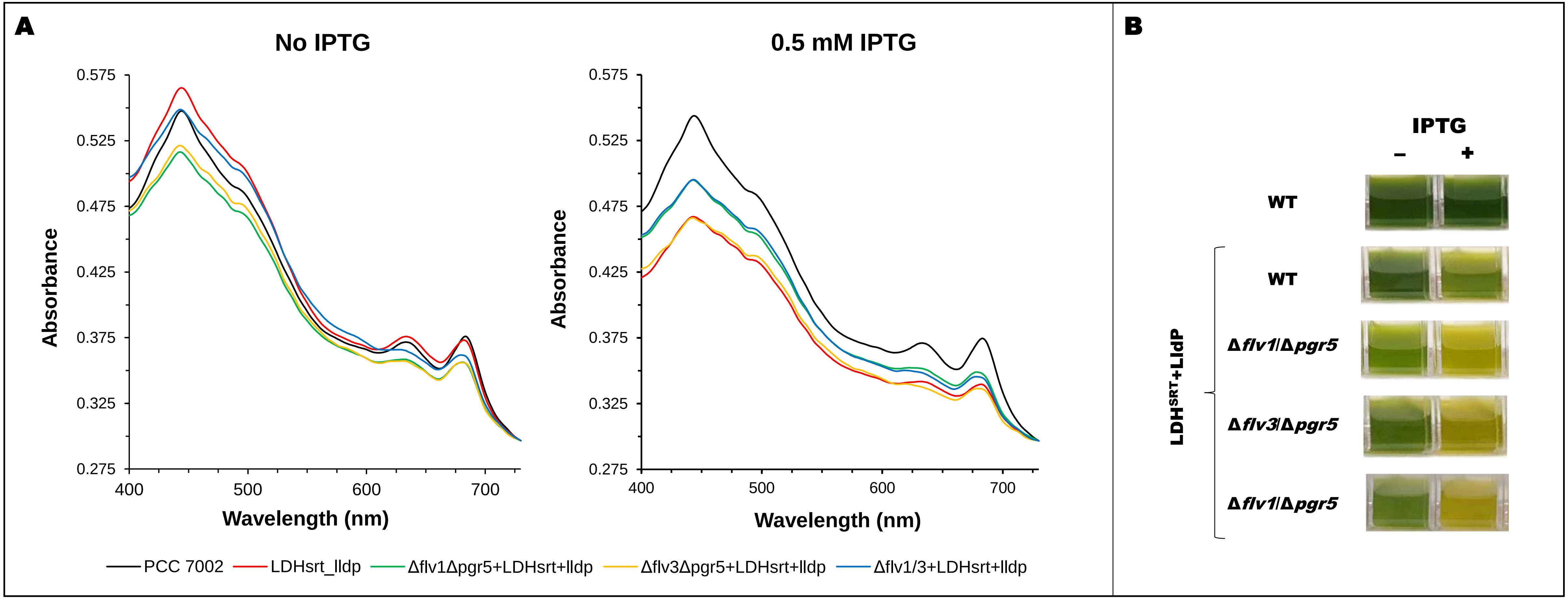

Figure S8

Pigmentation analysis of different D-lactate producing strains (WT background vs double knockout strains).

A) Whole cell absorbance spectra for PCC 7002 WT, cSJ008 (LDHsrt_lldP), cSJ075 (Δ*flv1*/Δ*pgr5* +LDHsrt_lldP), cSJ079 (Δ*flv3*/Δ*pgr5*+LDHsrt_lldP) and cSJ052 (Δ*flv1*/Δflv3+LDHsrt_lldP) strains after 5 days at 25 °C and 0.04% CO_2, in the absence (left panel) or presence (right panel) of 0.5 mM IPTG. All spectra were normalized relative to their absorbance at 730 nm.

B) Representative images of the same cultures. Pictures taken at the same time point.

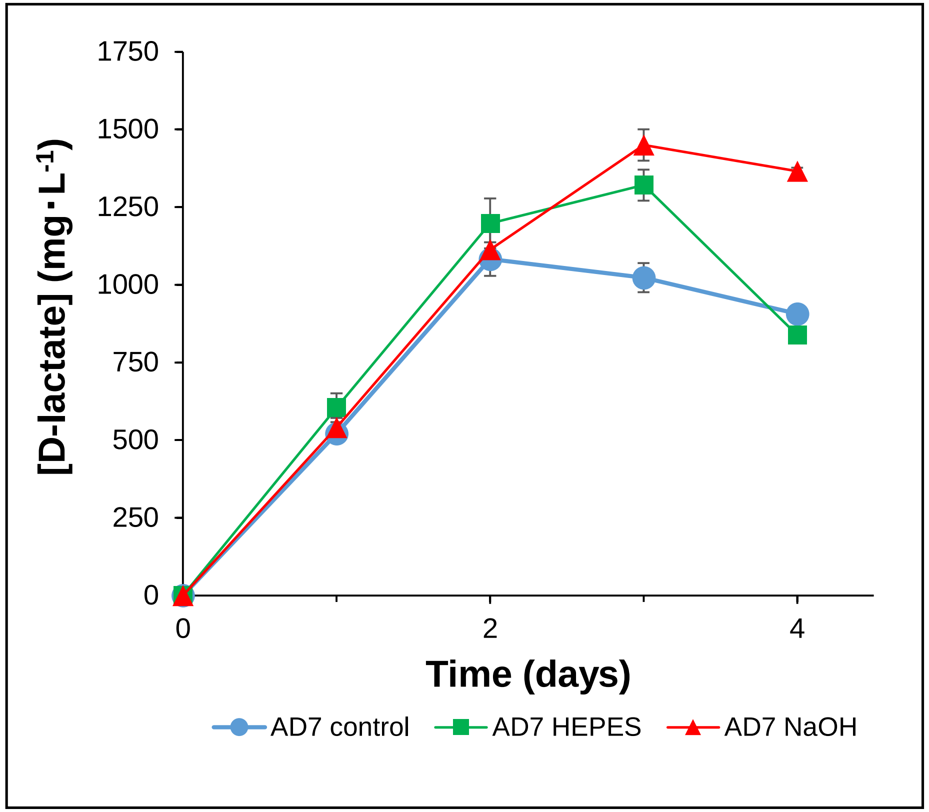

Figure S9 – D-lactate production in cSJ008 without or with different pH control methods. D-lactate concentrations in culture supernatants were measured at the time points indicated. “Control” cultures were only adjusted for water loss using sterile deionized water, “HEPES” cultures were supplemented with 50 mM HEPES, pH 8.0 at T=0 and pH of “NaOH” cultures was adjusted with a sterile 1M NaOH solution to pH=8.3 at T=2. Data is an average of 3 biological replicates, measured in technical duplicates. Error bars represent standard deviation (n=3).
